# Can optic nerve morphology in children with autism spectrum disorder be associated with atypical visual-sensory behaviors?

**DOI:** 10.3389/fpsyt.2025.1639695

**Published:** 2025-08-19

**Authors:** Mustafa Esad Tezcan, Abdullah Enes Ataş, Hurşit Ferahkaya

**Affiliations:** ^1^ Child and Adolescent Psychiatry, Selcuk Universitesi Tip Fakultesi, Konya, Türkiye; ^2^ Radiology, Necmettin Erbakan Universitesi Meram Tip Fakultesi Hastanesi, Konya, Türkiye; ^3^ Department of Child and Adolescent Psychiatry, Faculty of Medicine Hospital, Necmettin Erbakan University, Konya, Türkiye

**Keywords:** autism spectrum disorder, magnetic resonance imaging, optic nerve diameter, brain ventricles, corpus callosum, choroid plexus, optic chiasm

## Abstract

**Introduction:**

The aim of this study is to investigate, using magnetic resonance imaging (MRI), the optic nerve diameter, morphometric characteristics of the optic chiasm (OC), volumes of the lateral, third, and fourth ventricles, as well as the volumes of the corpus callosum (CC) and choroid plexus (CP) in children with autism spectrum disorder (ASD), and to compare these findings with those of a typically developing (TD) control group. Additionally, the study seeks to evaluate the impact of these neuroanatomical parameters on autism symptom severity and sensory sensitivity.

**Methods:**

This study included 111 children with ASD and 143 TD control children, aged between 5 and 13 years. The severity of ASD was assessed using the Social Communication Questionnaire (SCQ) and the Childhood Autism Rating Scale (CARS). Symptoms related to sensory sensitivities in ASD were evaluated using the Autism Behavior Checklist (AuBC).

**Results:**

In the ASD group, OC height, and the volumes of the CP and CC were significantly higher compared to the TD group, whereas OC width and third ventricular volume were significantly lower. There were no significant differences between the two groups in terms of optic nerve volumes, OC cross-sectional area, lateral and fourth ventricular volumes, or total brain volume. OC height was positively correlated with CARS, AuBC relationship, and AuBC use of body and objects scores, while OC width was positively correlated with CARS and AuBC use of body and objects scores. Conversely, OC height showed a negative correlation with AuBC personal-social development scores. After controlling for potential confounding variables such as total brain volume, age, and sex, the results of the covariance analysis remained unchanged. In multiple logistic regression analysis, left CP volume was found to be more strongly associated with ASD diagnosis compared to other morphometric measures.

**Discussion:**

The findings of this study suggest that increased OC height, increased CC and CP volumes, and decreased third ventricular volume may play a role in the etiopathogenesis of altered brain development in children with ASD.

## 1 Introduction

Autism spectrum disorder (ASD) is a lifelong neurodevelopmental disorder (NDD) characterized by impairments in social interactions, difficulties in verbal and nonverbal communication, repetitive behavior patterns, and restricted interests (1). In addition to the core symptoms, ASD is frequently associated with sensory processing dysfunctions, including visual processing deficits, which may underlie or exacerbate stereotyped behaviors observed in affected individuals (2, 3). A recent systematic review indicated that heightened visual and auditory sensitivities are linked to increased symptom severity in ASD (4). Atypical visual processing in ASD, including altered color perception, reduced eye contact, and impairments in gaze tracking, points to the potential importance of investigating retinal and optic nerve structures in understanding underlying neurobiological mechanisms (5). Furthermore, individuals with ASD have been reported to exhibit a higher prevalence of optic nerve hypoplasia and retinopathy compared to healthy controls. These visual impairments have been associated with deficits in depth perception and reduced peripheral visual processing, which may contribute to the social difficulties observed in ASD (6–8). Given their embryonic origin from the diencephalon, both the retina and optic nerve are integral components of the central nervous system and are frequently described as a ‘window to the brain’ due to their accessibility and neurodevelopmental relevance (9). Although certain components of the anterior visual pathway related to the retinal region can be easily assessed using optical coherence tomography (OCT), magnetic resonance imaging (MRI) may be required for comprehensive evaluation of the entire visual pathway. The volume or area of the optic chiasm has been reported as a potential MRI biomarker and a key component of the anterior visual pathway, as it can be measured using both automated and manual methods (10). Due to their neuroanatomical continuity with the brain, retinal and optic nerve measures have been utilized as structural indicators of axonal degeneration in conditions such as Alzheimer’s disease, multiple sclerosis, Parkinson’s disease, and NDDs (11).

NDDs are associated with structural abnormalities not only in the central nervous system (CNS) but also within subcortical regions, which are critical not only for motor control but also for higher-order functions such as learning, memory, attention, executive functioning, and emotion processing (12). The choroid plexus (CP) is a subcortical structure located in the lateral, third, and fourth ventricles of the brain. It serves as the primary source of cerebrospinal fluid (CSF) production, constitutes the core of the blood–CSF barrier, and plays a key role in maintaining brain homeostasis. In the context of volumetric alterations observed in patients with psychiatric disorders, assessment of CP volume—along with the bilateral lateral ventricles (LV), third, and fourth ventricles—is critically important for evaluating cognitive functions and brain development (13, 14). Neuroimaging analyses appear to be a useful approach for evaluating CP volume *in vivo*. In patients diagnosed with schizophrenia, morphological changes such as increased calcification in the CP have been reported using computed tomography (CT), and alterations in CP volume have also been demonstrated in various neuropsychiatric disorders using MRI (13, 15, 16). Increased volumes of the CP and lateral ventricles have been reported in individuals with Alzheimer’s disease, schizophrenia, bipolar disorder, and major depressive disorder. Moreover, CP abnormalities have been identified in pediatric cases of ASD, and animal studies have demonstrated ASD-like behaviors in mice with experimentally induced CP dysfunction (17, 18). The CP has been shown to interact with dopaminergic pathways, suggesting its involvement in processes related to learning and neuroplasticity. Additionally, CP enlargement has been linked to impairments in blood–brain barrier function and to neuroinflammatory activity (19, 20). Given the presence of neuroinflammation in ASD, reductions in neurotrophic factor levels in the CSF of children with ASD have been reported, potentially reflecting functional changes in the CP (14). Postmortem analyses in individuals with ASD, as well as findings from ASD animal models, have revealed elevated levels of proinflammatory cytokines in the CP, supporting the hypothesis that neuroinflammation may contribute to the etiopathogenesis of ASD (21).

The corpus callosum (CC), the brain’s largest commissural tract, plays a critical role in interhemispheric communication by integrating cortical and subcortical connections across multiple lobes. In the context of the atypical connectivity hypothesis in ASD, developmental differences in CC structure and function may represent a key neuroanatomical correlate of the disorder (22, 23). Studies have demonstrated smaller CC volumes in individuals with ASD, and ASD has been found to be more prevalent among individuals with agenesis of the CC (24). Recent meta-analytic and review studies have identified structural abnormalities in the CC tract in individuals with attention deficit hyperactivity disorder (ADHD) and ASD, particularly during the developmental transition from childhood to adulthood (25, 26). Findings from neuroimaging studies indicate that behavioral abnormalities observed in ASD may be attributed to disrupted functional connectivity across brain neural networks (27).

In this context, the development of MRI-based data for ASD represents a crucial step toward understanding the etiopathogenesis of such disorders through more detailed interpretation of neurobiological pathways, and for addressing the medical needs of children with ASD. In this study, optic nerve diameters and optic chiasm (OC) morphometry were examined as these structures reflect the integrity and functionality of the visual system during early development. It is proposed that these anatomical features may be associated with atypical visual perception and social interaction difficulties observed in ASD. The CC was included in the study due to its critical role in connectivity anomalies and impairments in cognitive-social integration reported in ASD. The volumes of the lateral, third, and fourth ventricles were assessed as potential indicators of neuroanatomical differences emerging during brain development. CP volumes were examined based on their involvement in ventricular system development and the hypothesis that they may play a role in neuroinflammatory processes increasingly associated with ASD. The aim of this study is to investigate the clinical relevance of these volumetric alterations in ASD and to determine how visual-sensory sensitivity and clinical symptomatology in individuals with ASD differ morphometrically from those in typically developing (TD) children. An additional question addressed in our cross-sectional study is the extent to which these macroscopic observational findings can predict an ASD diagnosis. Furthermore, atypical visual behavior and visual sensitivity observed in children with ASD were investigated through neuroimaging, with a focus on volumetric variations in the optic nerve and optic chiasm. Our hypothesis is that these regions—particularly the optic nerve and optic chiasm—may differ between children with ASD and TD children. We propose that the brain regions examined may be associated with symptoms related to sensory sensitivities and clinical severity in children with ASD. In our study, symptoms related to sensory sensitivities were assessed based on the individual’s responses to sensory inputs, as measured by the ‘Sensory’ and ‘Related Behaviors’ subscales of the Autism Behavior Checklist.

## 2 Materials and methods

### 2.1 Participants and procedures

**Figure 1:**
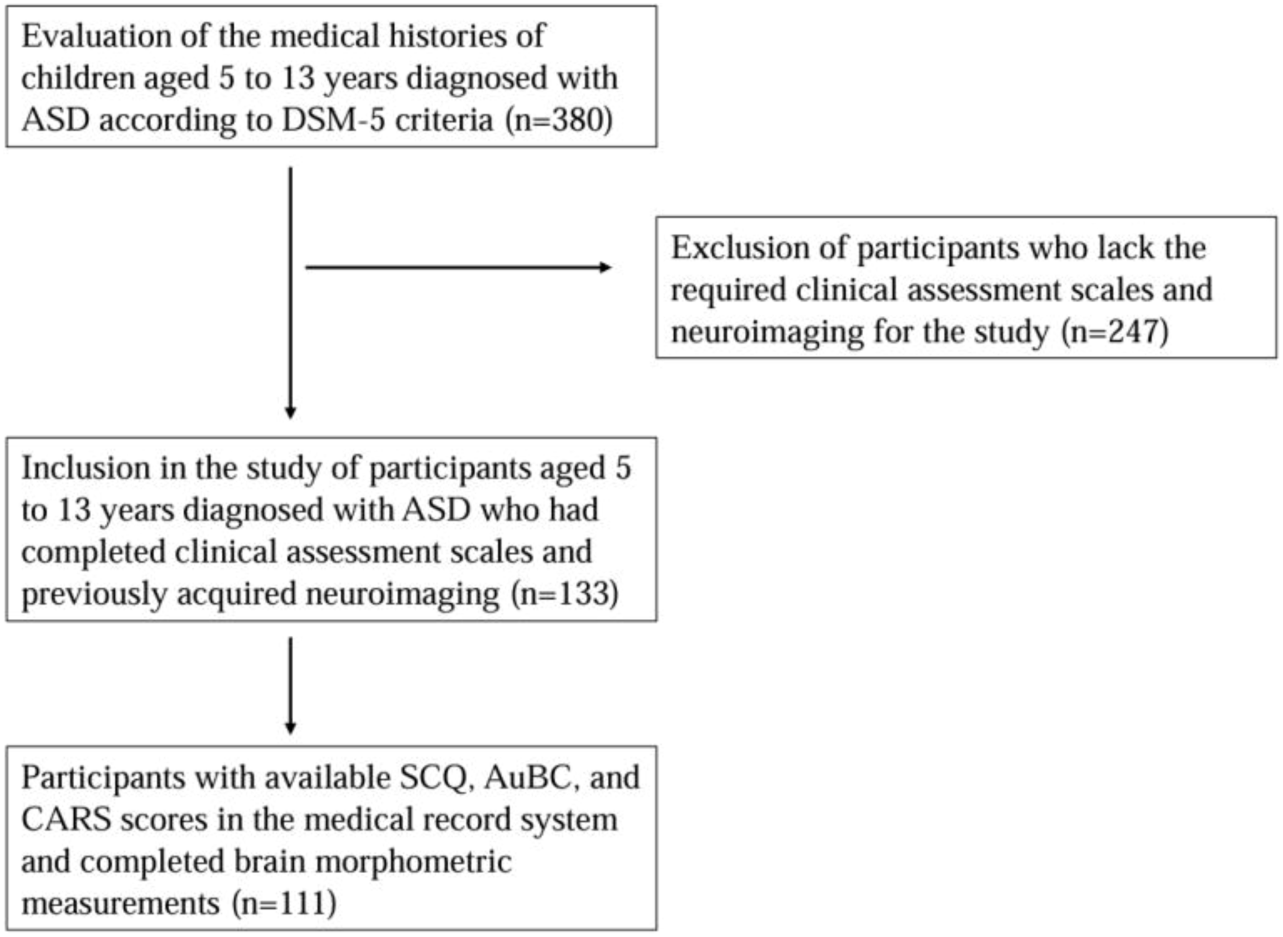
Flowchart of inclusion of participants with ASD, ASD, autism spectrum disorders; AuBC, autism behavior checklist; CARS, childhood autism rating scale; SCQ, social communication questionnaire; n, number.

Study participants were selected from children diagnosed with ASD at the Child and Adolescent Psychiatry Outpatient Clinic of Necmettin Erbakan University Faculty of Medicine Hospital, based on a review of the medical record system and the availability of appropriate neuroimaging data. Exclusion criteria included absence of neuroimaging, lack of clinical assessment scale data, history of organic brain injury or head trauma, postnatal intubation or mechanical ventilation, history of hypoxia-ischemia, known genetic disorders (including Down syndrome, Rett syndrome, Prader-Willi syndrome, Fragile X syndrome, and tuberous sclerosis), visual or hearing impairments, and chronic physical illnesses. Based on these inclusion and exclusion criteria, children diagnosed with ASD were deemed eligible and included in the study (**Figure 1**). In children diagnosed with ASD, brain MRI had previously been conducted as part of the assessment process to exclude underlying structural brain changes.

The TD control group consisted of children who had previously undergone brain MRI for various non-neurological and non-psychiatric reasons (e.g., vertigo or headache) but had not been diagnosed with any psychiatric, neurological, or physical disorders. The TD group was selected based on medical record review, and included only those who had been previously evaluated by a certified child psychiatrist. Participants without documented psychiatric evaluation in the medical record system were excluded from the study. MRI scans were performed by a specialist radiologist at the Radiology Department of Necmettin Erbakan University Faculty of Medicine Hospital. In this study, the TD children were selected from various neuroimaging procedures conducted at the same radiology unit, and all scans were obtained using the same structural imaging sequence. MRI data from both groups were retrospectively analyzed. A total of 111 children with ASD (aged 5–13 years) and 143 age- and sex-matched TD children were included in the study.

The study was conducted in accordance with the Declaration of Helsinki and was approved by the ethics committee of the Necmettin Erbakan University Faculty of Medicine (approval date: 25 April 2025, No. 2025/5727).

### 2.2 Diagnostic and symptom assessment

The children with ASD and TDs were evaluated with a certificated interview using the Schedule for Affective Disorders and Schizophrenia for School-Age Children, Present and Lifetime Version (K-SADS-PL), and ASD was diagnosed in accordance with the *Diagnostic and Statistical Manual of Mental Disorders, Fifth Edition*, criteria. The validity and reliability of the K-SADS-PL in the Turkish population was determined by Ünal et al. (28).

The Childhood Autism Rating Scale (CARS) is used to assess children older than 2 years with suspected autism and to differentiate children with autism from children with other developmental disorders (29). The scale, which has established validity and reliability in Turkish, consists of 15 items, each functioning as a subscale. Each item is rated on a scale from 1 to 4 in 0.5-point increments, resulting in a total score ranging from 15 to 60 (30). The Cronbach alpha value of the total score of the scale is 0.95.

The Autism Behavior Checklist (AuBC) was first used to describe the severity and frequency of autistic symptoms in school-age children and has been shown to be useful in young children (31). The scale consists of 57 items completed by parents and includes 5 subscales (sensorial stimulus, relationship, use of body and objects, language and personal-social development). Its validity and reliability in Turkey were determined by Yılmaz et al. (32). The cut-off score of the scale is 39, and the Cronbach alpha value of the total score of the scale is 0.92.

The Social Communication Questionnaire (SCQ), for which the Turkish version has demonstrated good reliability and validity (Cronbach’s alpha = 0.80), is a 40-item autism screening tool completed by the primary caregiver. Based on the items of the Autism Diagnostic Interview-Revised (ADI-R), the SCQ yields a total score ranging from 0 to 39. For nonverbal children, the total score ranges from 0 to 33. Each item assesses developmentally inappropriate behaviors, and higher scores are indicative of greater symptom severity (33, 34).

Evaluations in both groups were previously conducted by a specialist in child and adolescent psychiatry. For both groups, the clinician had completed sociodemographic data forms and recorded the medical history in the hospital’s electronic system. Following a review of the caregiver-reported history, the child’s developmental background and current functioning were assessed. A diagnosis of ASD was made in participants who met DSM-5 criteria, including at least two of four symptoms related to restricted and repetitive behaviors and all three symptoms related to deficits in social communication. The diagnosis was established by a qualified child psychiatrist using the DSM-5 and the K-SADS-PL. After the ASD diagnosis was confirmed, symptom severity was assessed using the SCQ, CARS, and AuBC, and the corresponding clinical scale scores were documented in the medical record system.

### 2.3 MRI procedures

**Figure 2:**
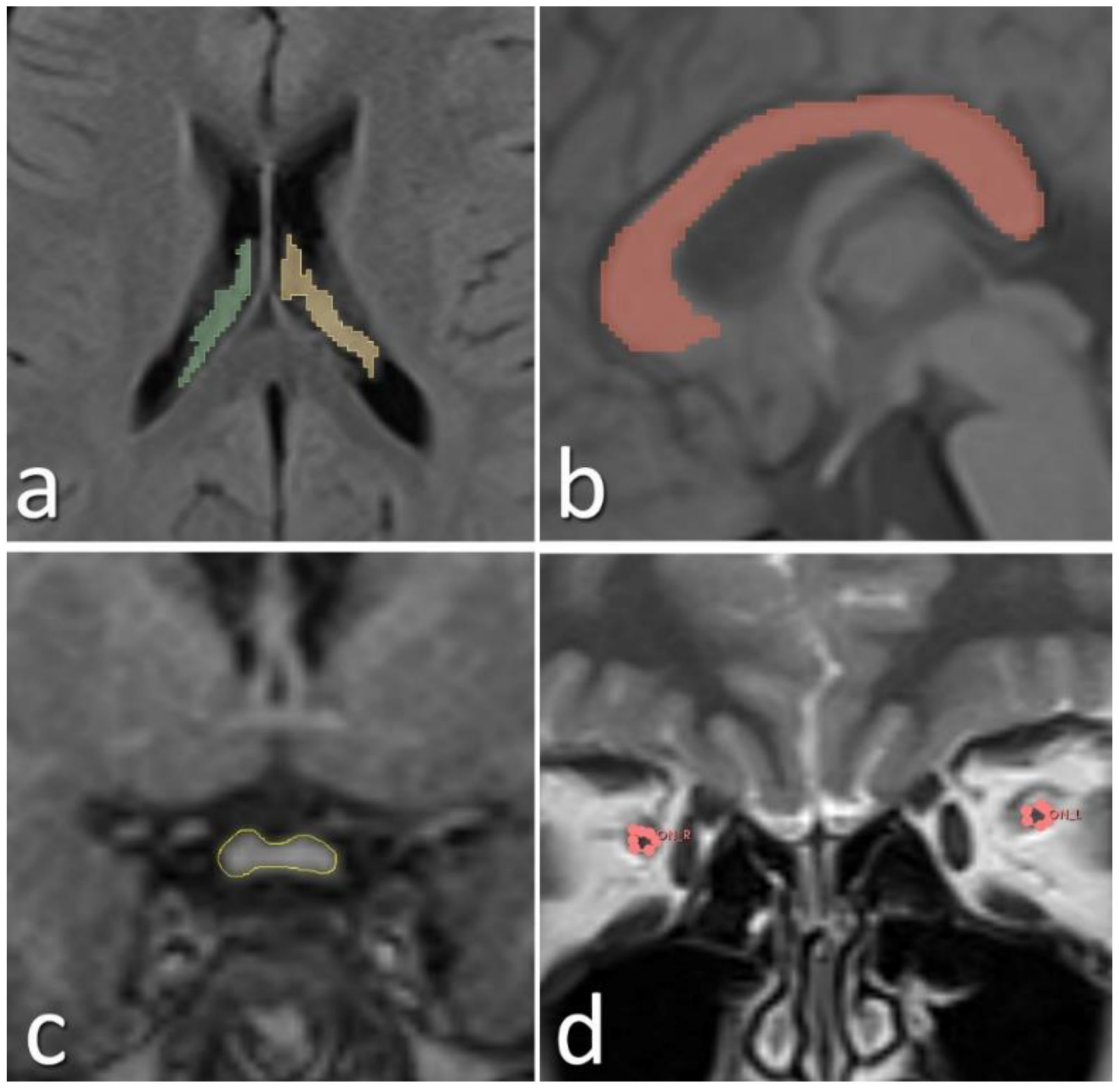
Manually segmented choroid plexus **(a)**, corpus callosum **(b)**, optic chiasm **(c)**, and optic nerves **(d)** from the acquired MR images are shown.

The scans were performed using the same scanner for both the ASDs and TD controls. MRI assessment was performed through repeated measurements by the same rater. All MRI scans were evaluated by an experienced radiologist. Prior to volumetric analyses, the radiologist was blinded to the study groups (participants with ASD and TD controls). Only age and sex information were provided to the evaluator; diagnostic group assignments were concealed. Blinding was achieved by randomly coding the MRI data and conducting the analyses independently of group identity. To assess the reliability of the MRI measurements, an intra-rater reliability analysis was conducted. The consistency between measurements was evaluated using the Intraclass Correlation Coefficient (ICC), yielding a value of 0.92. MRI images of the patients were acquired using a 1.5T MAGNETOM Aera MRI scanner (Siemens, Erlangen, Germany). The parameters for the MRI sequences utilized for the measurements were as follows: 3D isotropic T1 MPRAGE (slice thickness: 1 mm, matrix: 256x256, echo time: 3.14 ms, repetition time: 1520 ms, flip angle: 8 degrees, field of view: 250 mm), 2D FLAIR in the axial plane (slice thickness: 4 mm, matrix: 256x128, echo time: 92 ms, repetition time: 5300 ms, inversion time: 1875 ms, flip angle: 150 degrees, field of view: 250 mm), and T2 turbo spin echo in the coronal plane (slice thickness: 4 mm, matrix: 256x132, echo time: 92 ms, repetition time: 5120 ms, flip angle: 150 degrees, field of view: 250 mm). For reconstruction and volume measurement from the MR images, Syngo.via (Siemens, Erlangen, Germany) and the open-source 3D-Slicer applications were employed (35). Segmentation of the measured volumes was performed manually (**Figure 2**).

### 2.4 Statistical analyses

The Python programming language (Python Software Foundation, https://www.python.org/) was used for multiple logistic regression analysis, while statistical analyses were performed using SPSS version 26.0 (SPSS Inc., Chicago, IL). Depending on the distribution characteristics, age and brain morphometric parameters were compared between the two groups using either the Student’s *t*-test or the Mann–Whitney *U* test. The chi-square test was used to assess the frequency distribution of categorical variables between groups. Normal distribution was determined based on skewness and kurtosis values, with values between −2 and +2 considered acceptable (36). A significance level of p < 0.05 was accepted within a 95% confidence interval. To minimize the risk of Type II error due to multiple variables, multivariate analyses were performed. Age, total brain volume, and sex were included as covariates, and brain morphometric parameters were compared between groups using multivariate analysis of covariance (MANCOVA). The volumes of the third ventricle were logarithmically transformed to achieve normal distribution. Since the MANCOVA test revealed a significant difference between the groups, follow-up univariate analyses of covariance (ANCOVA) were conducted for each outcome variable.

Effect sizes for both parametric and non-parametric comparisons were estimated using Cohen’s *d* and Cramér’s *V* for categorical variables. According to conventional benchmarks, Cohen’s *d* values ≥ 0.8 were considered large, 0.5–0.7 intermediate, 0.2–0.4 small, and < 0.2 no effect (37, 38). Correlations between brain morphometric measurements and clinical variables were assessed using Pearson’s correlation for parametric data and Spearman’s correlation for non-parametric data.

In addition, a multiple logistic regression model was applied to evaluate the potential effects of morphometric variables on the ASD diagnosis. Considering the Cohen’s *d* effect size, sample sizes, alpha level (α=0.05) and intergroup sample ratio, *post-hoc* power was calculated for each morphometric measurement.

## 3 Results

A total of 254 participants were included in the study, comprising 111 children diagnosed with ASD and 143 typically developing (TD) controls. No statistically significant differences were observed between the two groups in terms of age or sex (*t* = -1.170, *p* = 0.243 and *x^2^* = 3.482, *p* = 0.062, respectively). Demographic characteristics, SCQ, CARS, and AuBC scores, as well as brain morphometric measurements of participants in both groups, are presented in **Table 1**.

**Table 1:** Data on brain morphometric measurements, SCQ, CARS, and AuBC scales, as well as the demographic characteristics of participants in both groups.

	ASD (n: 111)	TD (n: 143)	*p*	*t*/*z/x^2^*	*d*
Age (years)	7.98± 4.01	8.53 ± 3.46	0.243	-1.170	0.146
Sex	Girl (24) Boy (87)	Girl (46) Boy (97)	0.062	3.482	0.117^a^
Right optic nerve diameter (mm)	2.61 ± 0.41	2.63 ± 0.42	0.694	-0.393	0.049 0.06^b^
Left optic nerve diameter (mm)	2.61 ± 0.40	2.66 ± 0.39	0.274	-1.096	0.138 0.19^b^
Optic chiasm height (mm)	2.64 ± 0.55	2.39 ± 0.44	**<0.001**	3.964	**0.494** 0.97^b^
Optic chiasm width (mm)	11.79 ± 1.40	12.17 ± 1.46	**0.042**	-2.041	0.025 0.53^b^
Optic chiasm cross-sectional area (mm^2^)	27.49 ± 7.93	25.86 ± 5.47	0.055	1.930	0.023 0.46^b^
Right CP volume (cm^3^)	1.33 ± 0.41	1.04 ± 0.43	**<0.001**	5.402	**0.685** 1.0^b^
Left CP volume (cm^3^)	1.39 ± 0.47	0.99 ± 0.36	**<0.001**	7.659	**0.952** 1.0^b^
Right lateral ventricle volume^c^ (cm^3^)	6.31 ± 5.18	5.30 ± 4.48	0.059	-1.887	0.209 0.37^b^
Left lateral ventricle volume^c^ (cm^3^)	6.56 ± 6.51	5.56 ± 5.37	0.066	-1.837	0.168 0.26^b^
Third ventricle volume^c^ (cm^3^)	0.60 ± 0.40	0.68 ± 0.35	**0.001**	-3.221	0.199 0.34^b^
Fourth ventricle volume (cm^3^)	1.59 ± 0.64	1.54 ± 0.47	0.478	0.710	0.088 0.10^b^
CC volume (cm^3^)	3.55 ± 1.01	3.32 ± 0.72	**0.037**	2.100	0.260 0.53^b^
Total brain volume (cm^3^)	1171.76 ± 137.70	1194.89 ± 113.32	0.143	-1.468	0.018 0.30^b^
CARS	33.35 ± 6.70	–	–	–	–
AuBC sensorial stimulus	7.92 ± 3.09	–	–	–	–
AuBC relationship	28.05 ± 5.12	–	–	–	–
AuBC use of body and objects	29.56 ± 5.18	–	–	–	–
AuBC language	22.04 ± 6.03	–	–	–	–
AuBC personal-social development	15.46 ± 2.53	–	–	–	–
SCQ Total score	31.25 ± 3.64	–	–	–	–

ASD, autism spectrum disorder; TD, typically developing control; CP, choroid plexus; CC, corpus callosum; AuBC, autism behavior checklist; CARS, childhood autism rating scale; SCQ, social communication questionnaire; d, Cohen’s d effect size.

a Cramér’s V effect size,

b Post-hoc power analysis,

c Mann-Whitney U.

Bold values indicate statistically significant results.

Optic chiasm height, right and left choroid plexus volumes, and corpus callosum volume were significantly higher in the ASD group compared to the TD group (*t* = 3.964, *p* < 0.001; *t* = 5.402, *p* **<** 0.001; *t* = 7.659, *p* **<** 0.001; *t* = 2.100, *p* = 0.037, respectively). In contrast, optic chiasm width and third ventricle volume were significantly lower in the ASD group compared to the TD group (*t* = -2.041, *p =* 0.042; *z* = -3.221, *p* **=** 0.001, respectively). No significant differences were found between the two groups in terms of optic nerve volumes, optic chiasm cross-sectional area, lateral ventricle volumes, fourth ventricle volume, or total brain volume. Left CP volume was observed to have a large effect sizes (Cohen’s *d*= 0.952). *Post-hoc* power analyses related to the brain morphometric measurements are presented in **Table 1**.

**Figure 3:**
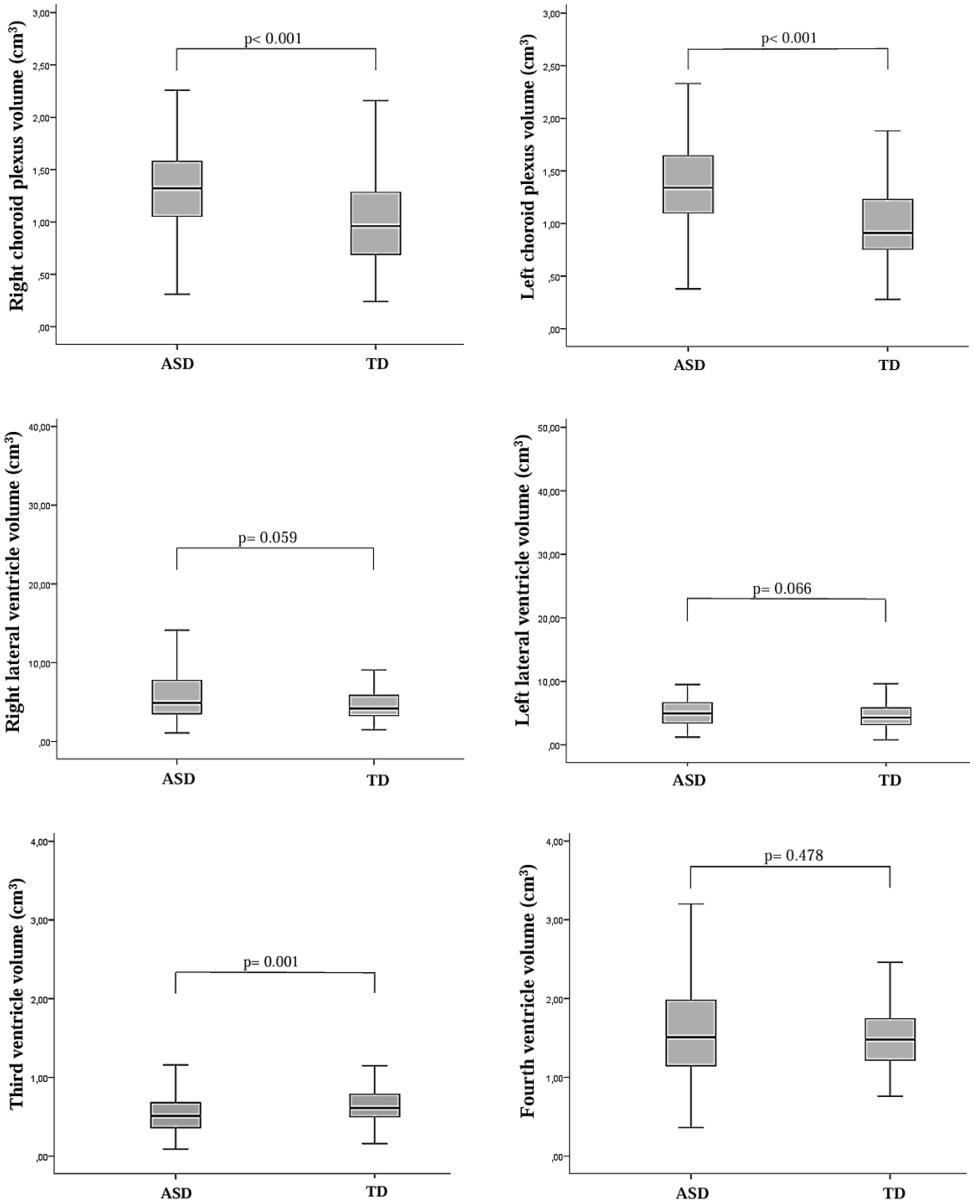
Box plots illustrating the distribution of total choroid plexus and lateral ventricle volumes, as well as third and fourth ventricle volume levels, in children diagnosed with autism spectrum disorder (ASD) and typically developing (TD) control children. The Mann–Whitney *U* test was used to compare lateral ventricle volumes and third ventricle volume levels between the two groups.

**Figure 4:**
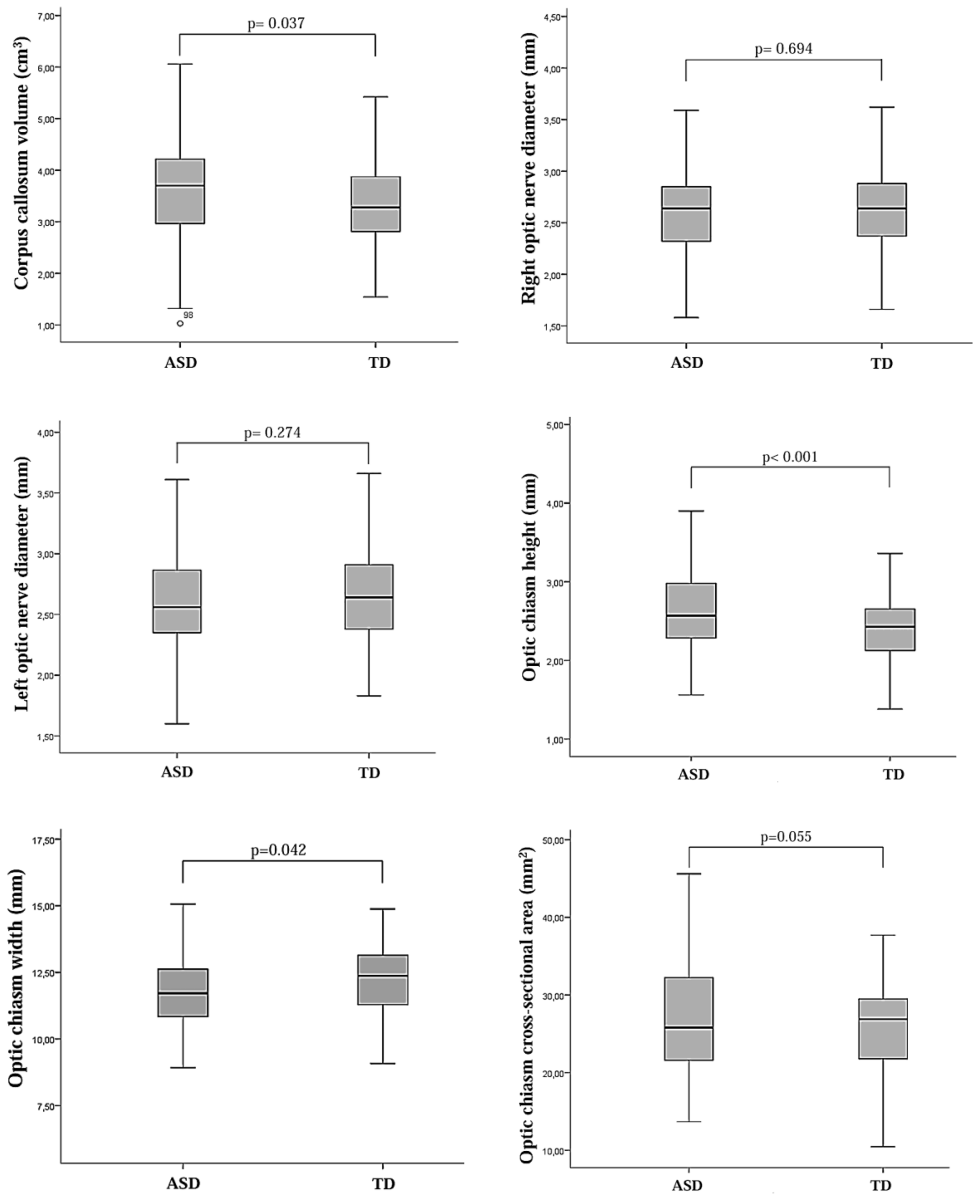
Box plots showing the distribution of morphometric measurements of the optic nerve, optic chiasm, and corpus callosum in children diagnosed with autism spectrum disorder (ASD) and typically developing (TD) control children.

**Figure 5:**
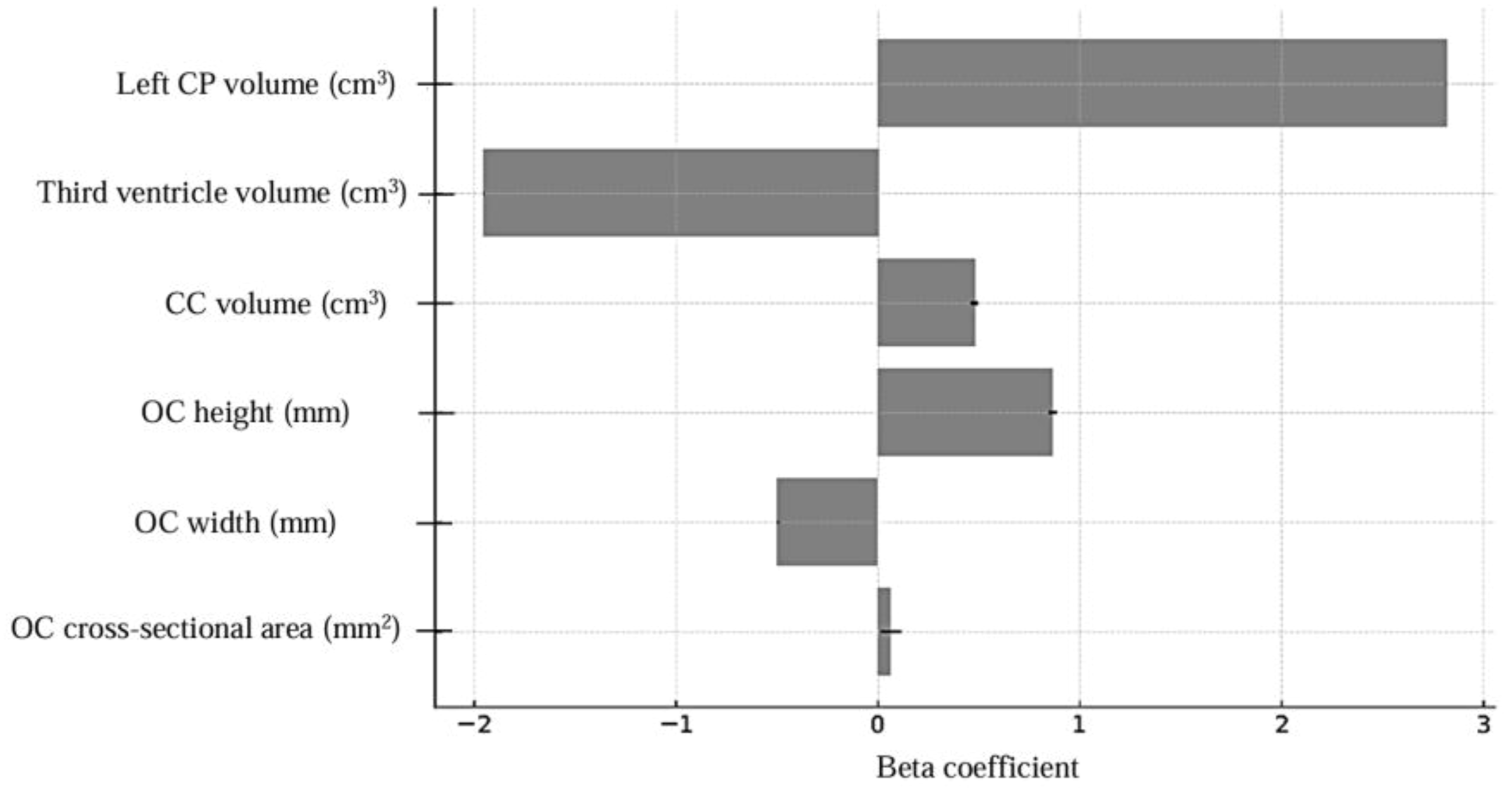
Multiple logistic regression model coefficients, CP, choroid plexus; CC, corpus callosum; OC, optic chiasm.

Brain MRI parameters for both groups are presented in **Figures 3** and **4**. In the multiple logistic regression analysis, left CP volume (β = 2.82, p < 0.001), third ventricle volume (β = -1.95, p = 0.001), CC volume (β = 0.48, p = 0.018), OC height (β = 0.87, p = 0.020), OC width (β = -0.49, p = 0.002), and OC cross-sectional area (β = 0.063, p = 0.050) were identified as statistically significant predictors of ASD diagnosis. The model’s log-likelihood value was -0.470 (p < 0.05). Among these, left choroid plexus volume was observed to be the strongest predictor (**Figure 5**).

A MANCOVA was performed to avoid type II errors caused by the multi-test effect and to control for confounding factors such as total brain volume, sex and age. The MANCOVA revealed a significant difference between the groups [Pillai’s trace *V* = 0.357, *F*_(6.244)_ = 22.549, *p* < 0.001, ηp^2^ = 0.357]. The same variables were taken as covariates in the ANCOVA to determine the variables that caused the differences between the groups. The results were unchanged, and the OC height [*F*_(1.249)_ = 16.121, *p* < 0.001, ηp^2^ = 0.069], right CP volume [*F*_(1.249)_ = 32.814, *p* < 0.001, ηp^2^ = 0.235], left CP volume [*F*_(1.249)_ = 66.821, *p* < 0.001, ηp^2^ = 0.297], and CC volume [*F*_(1.249)_ = 5.373, *p* = 0.021, ηp^2^ = 0.089] were significantly higher, whereas the OC width [*F*_(1.249)_ = 2.591, *p* = 0.109, ηp^2^ = 0.209] and log-third ventricle volume [*F*_(1.249)_ = 11.487, *p* = 0.001, ηp^2^ = 0.086] were significantly lower. The ANCOVA results, after controlling for total brain volume, sex, and age, are presented in **Table 2**.

**Table 2:** Comparison of OC, CP, CC, and third ventricle volume levels between the two groups according to ANCOVA.

Total samples ANCOVA^a^	ASD (n: 111)		TD (n: 143)		*F* (1.249)	*p*	η_p_^2^	Observed power
	Mean	SD	Mean	SD				
OC height	2.64	0.55	2.39	0.44	16.121	**< 0.001**	0.069	0.979
OC width	11.79	1.40	12.17	1.46	2.591	**0.109**	0.209	0.361
Right CP volume	1.33	0.41	1.04	0.43	32.814	**< 0.001**	0.235	1.000
Left CP volume	1.39	0.47	0.99	0.36	66.821	**< 0.001**	0.297	1.000
CC volume	3.55	1.01	3.32	0.72	5.373	**0.021**	0.089	0.636
Log-Third ventricle volume	-0.29	0.26	-0.20	0.18	11.487	**0.001**	0.086	0.922

CP, choroid plexus; CC, corpus callosum; OC, optic chiasm; ANCOVA, analysis of covariance; ASD, autism spectrum disorder; TD, typically developing controls.

a Covariates: total brain volume, age and sex.

Bold values indicate statistically significant results.

In the ASD group, correlations between SCQ, CARS, and AuBC scale scores and brain morphometric parameters were assessed using Pearson’s and Spearman’s correlation tests. Right lateral ventricle and fourth ventricle volumes showed weak positive correlations (*r*= 0.193, *p*= 0.042; *r*= 0.227, *p*= 0.017, respectively) with the total SCQ score, while right and left optic nerve diameters were weak positively correlated with the AuBC sensorial stimulus scores (*r*= 0.190, *p*= 0.045; *r*= 0.204, *p*= 0.032, respectively). OC height and OC cross-sectional area were weak positively correlated with CARS (r = 0.289, p = 0.002; r = 0.300, p = 0.001, respectively), AuBC relationship scores (r = 0.314, p = 0.001; r = 0.262, p = 0.006, respectively), and AuBC use of body and objects scores (r = 0.307, p = 0.001; r = 0.248, p = 0.009, respectively). Additionally, OC width showed weak positive correlations with CARS and AuBC use of body and objects scores (*r*= 0.271, *p*= 0.004; *r*= 0.306, *p*= 0.001, respectively). However, OC height showed a weak negative correlation with the AuBC personal-social development score, and OC cross-sectional area was weak negatively correlated with the AuBC language score (*r*= -0.208, *p*= 0.028; *r*= -0.230, *p*= 0.015, respectively). No correlation was found between the other variables (**Table 3**).

**Table 3:** Correlation coefficients between brain morphometric measurement levels and CARS, SCQ and AuBC scores.

Brain Morphometric Measurements		CARS	SCQ	AuBC sensorial stimulus	AuBC relationship	AuBC use of body and objects	AuBC language	AuBC personal-social development
Right CP	*p* *r*	0.560 0.056	0.335 -0.092	0.712 -0.035	0.926 -0.009	0.276 0.104	0.994 -0.001	0.694 0.038
Left CP	*p* *r*	0.415 0.078	0.536 -0.059	0.634 -0.046	0.465 -0.070	0.471 0.069	0.407 0.080	0.721 0.034
Right LV	*p* *r*	0.475 0.069	**0.042** **0.193**	0.971 0.004	0.118 -0.149	0.114 0.151	0.750 -0.031	0.461 0.071
Left LV	*p* *r*	0.665 0.042	0.061 0.178	0.278 0.104	0.431 -0.075	0.171 0.131	0.986 0.002	0.576 0.054
Third ventricle	*p* *r*	0.844 0.019	0.074 0.170	0.778 0.027	0.167 -0.132	0.425 0.076	0.966 -0.004	0.703 -0.037
Fourth ventricle	*p* *r*	0.910 0.011	**0.017** **0.227**	0.551 0.057	0.792 -0.025	0.242 0.112	0.321 0.095	0.106 0.154
CC	*p* *r*	0.751 -0.030	0.822 0.022	0.392 0.082	0.513 -0.063	0.427 -0.076	0.319 0.095	0.829 -0.021
Right optic nerve diameter	*p* *r*	0.865 0.016	0.061 0.179	**0.045** **0.190**	0.751 -0.030	0.636 -0.045	0.565 -0.055	0.669 0.041
Left optic nerve diameter	*p* *r*	0.479 -0.068	0.129 0.145	**0.032** **0.204**	0.372 -0.086	0.380 -0.084	0.955 -0.005	0.318 0.096
OC height	*p* *r*	**0.002** **0.289**	0.656 -0.043	0.781 -0.027	**0.001** **0.314**	**0.001** **0.307**	0.065 -0.176	**0.028** **-0.208**
OC width	*p* *r*	**0.004** **0.271**	0.825 0.021	0.362 -0.087	0.193 0.124	**0.001** **0.306**	0.249 -0.110	0.889 -0.013
OC cross-sectional area	*p* *r*	**0.001** **0.300**	0.960 -0.005	0.156 -0.135	**0.006** **0.262**	**0.009** **0.248**	**0.015** **-0.230**	0.160 -0.134

CP, choroid plexus; CC, corpus callosum; OC, optic chiasm; LV, lateral ventricle; AuBC, autism behavior checklist; CARS, childhood autism rating scale; SCQ, social communication questionnaire.

Bold values indicate statistically significant results.

## 4 Discussion

To the best of our knowledge, this study is the first to investigate optic nerve and optic chiasm morphometry using MRI in children diagnosed with ASD, as well as the relationship between these morphometric differences and ASD symptom severity. Optic chiasm height, right and left choroid plexus volumes, and corpus callosum volumes were significantly higher in the ASD group compared to the TD group (regardless of total brain volume, sex and age). Optic chiasm width and third ventricle volume were significantly lower in the ASD group compared to the TD group, regardless of total brain volume, sex, and age. No significant differences were found between the two groups in optic nerve volumes, optic chiasm cross-sectional area, lateral ventricle volumes, fourth ventricle volume, or total brain volume. Multiple logistic regression analysis revealed that left choroid plexus volume was more strongly associated with ASD diagnosis compared to other morphometric variables.

### 4.1 Optic nerve morphometry in ASD

In our study, the height levels of the optic chiasm were significantly higher in the ASD group compared to the TD group, whereas the width of the optic chiasm was significantly lower in the ASD group than in the TD group. A review of the literature shows that visual studies related to ASD have predominantly focused on the retinal layers rather than the optic nerve. In line with this, it has been reported that children with ASD exhibit significantly reduced average choroidal thickness and volumes of the ganglion cell layer and inner plexiform layer in both eyes compared to controls 9. In an optical coherence tomography (OCT)-based study, participants with ASD demonstrated higher optic nerve head (ONH) perfusion density in the peripapillary inferior quadrant, greater macular thickness, increased peripapillary retinal nerve fiber layer (pRNFL) thickness in the inferior clock-hour sectors, and higher macular vessel density compared to TDs (39). In adults diagnosed with ASD, reduced macular and outer nuclear layer thickness compared to controls has also been reported, and decreased macular thickness has been found to be significantly and inversely associated with the severity of autistic symptoms (40). Using OCT, a significantly increased thickness of the ellipsoid zone, where cone photoreceptors are located, has been observed in children with ASD compared to TDs (5). The RNFL, primarily composed of ganglion cell axons that synapse directly with the lateral geniculate nucleus (LGN), may represent an appropriate region for analysis in CNS studies (41). Moreover, ganglion cells, whose axons form the optic nerve, have been shown to be particularly vulnerable to damage and neurodegeneration (42). A recent meta-analysis reported that patients with schizophrenia spectrum disorders exhibit significantly reduced ganglion cell layer thickness compared to healthy controls. Furthermore, it has been observed that the thickness of these layers may be influenced by antipsychotic medication use (43, 44). Patients with major depressive disorder have been reported to exhibit significantly reduced RNFL and macular thickness compared to healthy controls, and this thinning has been associated with both sleep quality and the severity of depressive symptoms (45). A reduction in RNFL thickness has been observed to be associated with neuroinflammation and neurodegeneration (46).

A recent review also noted that pale-appearing optic discs in children diagnosed with ASD are often associated with optic nerve atrophy or hypoplasia, suggesting that monitoring the optic nerve may be useful for assessing potential progressive neurodegeneration in ASD (47). White matter abnormalities associated with atypical myelination have been reported in ASD, and such disruptions may impair optic nerve development. Additionally, the optic pit originates from the forebrain during embryonic development. Abnormal brain development in individuals with autism may lead to visual impairments related to the neural retina. Furthermore, the development of the optic nerve and neural retina can be disrupted due to the loss of oligodendrocytes, which are responsible for myelination. Myelin expression in oligodendrocytes is regulated by WNT/β-catenin signaling, a pathway that has been reported to be altered in ASD (6, 48, 49). In ASD animal models induced by valproic acid, it has been observed that valproic acid can cause developmental delays in the formation of retinal neurons by affecting the WNT signaling pathway, which in turn may influence visual behaviors (50, 51). Approximately 30% of children diagnosed with optic nerve hypoplasia and septo-optic dysplasia have been reported to exhibit comorbid ASD (52). In relation to the optic nerve, the retinal layer has been reported to initially thicken due to early neuroinflammation, followed by thinning associated with subsequent neurodegeneration (53). Neuroinflammatory processes observed in ASD have been reported to lead to neuronal damage and disruption of synaptic connectivity, which may contribute to the progression of clinical symptoms (54). In our study, the observed increase in optic chiasm height and decrease in optic chiasm width in ASD were not clearly demonstrated to be related to neuroinflammation or age-related changes. The reduced optic chiasm width may be associated with retinal layer thinning observed in ASD; however, the nature of this relationship remains unclear. Considering that retinal and optic nerve morphometry may vary depending on factors such as age and psychotropic medication use, further studies with larger sample sizes, inclusion of individuals on medication, and the use of advanced analytical methods such as OCT are needed to better understand the association of this finding with ASD.

In our study, OC height levels showed a weak positive correlation with CARS scores, AuBC relationship scores, and AuBC use of body and objects scores, while OC width levels were weak positively correlated with CARS and AuBC use of body and objects scores. Conversely, OC height exhibited a weak negative correlation with AuBC personal-social development scores. Previous studies investigating the relationship between the visual system and clinical symptomatology in ASD have primarily focused on retinal alterations. In this context, various studies have reported that retinal layer abnormalities observed in ASD are associated with increased symptom severity (6, 9, 40, 55). Abnormal development of the visual cortex in the early stages of ASD suggests the possibility of disrupted visual information transmission from retinal ganglion cells to the visual cortex (56). In ASD, retinal dysfunction has been observed to be accompanied by cortical functional impairments, and this has been linked to various social and communicative difficulties associated with the disorder (57, 58). In individuals with ASD who exhibit marked impairments in verbal intelligence, a thinner RNFL has been demonstrated (41). In a study focusing on the olfactory bulb, which is anatomically adjacent to the optic nerve, olfactory bulb volumes in individuals with ASD were reported to show a positive correlation with AuBC use of body and objects scores (59). The retina and cerebral cortex both originate from the embryonic prosencephalon, one of the three primary brain vesicles. During the early stages of neurodevelopment, the prosencephalon differentiates into the optic vesicles—which will give rise to the retina and optic nerve—and the telencephalic vesicles, which will later develop into various structures including the cerebral cortex (60). The retina is often referred to as a ‘window to the brain’ due to its embryological origin from the same germ layer as the brain and its cellular similarities. As such, changes occurring in the brain can potentially be inferred through direct observation of the retina (44). A recent review reported that clinical features observed in children diagnosed with ASD are also present in children with visual impairments, and that the visual system shows a positive correlation with social-communicative skills. According to the AuBC, the prevalence of an ASD diagnosis among children with visual impairments was found to be 23.5%. In visually impaired children, language and communication difficulties may arise as a consequence of visual deficits (61). The variability in optic chiasm measurements observed in ASD in our study may be related to abnormal sensory sensitivities, as suggested by previous research. This finding highlights the need for further longitudinal studies incorporating MRI and other advanced neuroimaging techniques to clarify the relationship between optic chiasm alterations, clinical symptoms, and atypical sensory processing in ASD. To gain a better understanding of visual impairments in this population, future studies should include detailed evaluations of both the optic nerve and retinal layers. Moreover, further research utilizing objective or subjective tools for assessing sensory characteristics (e.g., Sensory Profile 2) is needed to more clearly investigate the relationship between the diameters of the right and left optic nerves and sensory response scores on the AuBC.

### 4.2 Choroid plexus and ventricular volumes in ASD

In our study, right and left CP volumes were significantly higher in the ASD group compared to the TD group. To the best of our knowledge, only two MRI studies have investigated CP volume in ASD. A large-scale retrospective analysis including structural brain MRIs of 1,769 individuals with ASD and 1,996 TD controls (aged 0–32 years) reported increased CP and ventricular volumes in the ASD group (62). Another study also reported abnormalities in the left choroid plexus in individuals with ASD compared to TD controls (63). The volumetric enlargement of the choroid plexus observed in ASD has been reported to be potentially associated with increased levels of inflammatory cytokines in the CSF (64). Increased choroid plexus and ventricular volumes have also been reported in patients with schizophrenia spectrum disorders and major depressive disorder compared to healthy controls (13, 65, 66). The CP, which plays a role in cerebrospinal fluid (CSF) production, has also been observed to synthesize cytokines, neurotrophic factors, and peptides such as insulin-like growth factor (IGF) (18). Increased CSF volume has been reported in individuals with ASD compared to TD controls, and this has been suggested to be associated with impaired neurogenesis and heightened neuroinflammatory activity in the choroid plexus (67). Several studies have reported elevated levels of proinflammatory cytokines as well as increased IGF-1 and IGF-2 concentrations in the CSF of individuals with ASD compared to controls (14, 68, 69). As reported in a meta-analysis, neuroinflammatory activity in ASD has been observed not only in the CSF but also in peripheral blood (70). The increased CP volumes observed in our study in individuals with ASD are consistent with previous findings. However, neuroinflammation was not assessed and CSF analysis was not performed in our study. Given that CP enlargement in ASD may vary with age and be influenced by underlying neuroinflammatory processes, future studies should include more detailed evaluations of CSF and markers of neuroinflammation. In this context, advanced analyses integrating CSF, peripheral blood biomarkers, and CP volumetric measurements are warranted to better understand their interrelations and relevance to ASD.

In the regression analysis conducted in our study, the volume of the left choroid plexus was found to show a stronger association with the ASD diagnosis compared to other morphometric variables. A review of the literature suggests that this finding may be related to alterations in hemispheric dominance observed in individuals with ASD. A recent review reported greater brain activity in the left hemisphere compared to the right in individuals with ASD, which has been hypothesized to result from the left hemisphere exhibiting less atypical development than the right in ASD (71). In addition, an asymmetric increase in gray matter volume in the left postcentral gyrus — corresponding to the primary somatosensory cortex — has been reported in individuals with ASD (72). Functional neuroimaging studies have reported altered lateralization during language-related tasks in both children and adults with ASD, characterized by reduced left-hemisphere dominance and increased rightward asymmetry (73). An age-related increase in hemispheric asymmetry has also been reported in individuals with ASD (74). The association between left CP volume and ASD observed in our study may reflect atypical lateralization related to altered hemispheric dominance in ASD (75). However, asymmetry between the right and left CP morphometry could not be assessed in our study, and therefore could not be linked to ASD. Additionally, differences between left and right CP volumes were not examined. A review of the literature suggests that asymmetry measurements of the CP may hold potential significance. To more clearly demonstrate the relationship between our findings and lateralization in ASD, longitudinal studies investigating both hemispheric asymmetry and interhemispheric differences in CP volumes are warranted.

In our study, third ventricular volume was significantly lower in the ASD group compared to the TD group, whereas no significant differences were found between the groups in terms of lateral and fourth ventricular volumes. In contrast, several studies have reported larger lateral, third, and fourth ventricular volumes in individuals with ASD compared to TDs (62, 76–78). Additionally, significant asymmetrical differences in ventricular volumes have been reported in individuals with ASD compared to TDs (79). A longitudinal study reported that ASD participants with a history of prenatal hypoxic exposure had larger third ventricular volumes compared to both ASD participants without such exposure and TD controls. Furthermore, significant associations were found between prenatal hypoxic exposure and the severity of sensory dysfunction and sleep disturbances (80). In patients with first-episode psychosis and pediatric-onset bipolar disorder, lateral and third ventricular volumes have been reported to be significantly larger compared to healthy controls, and antipsychotic medication use has been suggested to influence ventricular volüme (81, 82). A reduction in ventricular volumes associated with antidepressant use has also been reported in various psychiatric disorders (83). Newly produced CSF flows from the lateral, third, and fourth ventricles into the brain cisterns and subsequently enters the subarachnoid space, covering the outer cortical surfaces of the brain. Disruptions in CSF circulation may affect the ventricles and contribute to the development of neuroinflammation (84). A recent review reported increases in 26 inflammatory cytokines in the CSF of individuals with ASD, along with elevated CSF volumes. The increase in CSF volume has been associated with ventricular enlargement, which may exert pressure on brain tissue and potentially lead to neural damage. Furthermore, CSF and ventricular volumes in ASD have been shown to be elevated compared to TD children up to the age of two, with a tendency to decrease with age (12, 85). Neuroinflammation has been shown to affect ventricular volume through increased microglial activation and elevated cytokine levels (86). The reduced third ventricular volumes observed in our study in individuals with ASD are not consistent with previous findings. This finding may vary depending on factors such as age range, psychotropic medication use, neuroimaging protocols, and segmentation techniques. Furthermore, in our study, inflammatory status could not be evaluated either centrally or peripherally in relation to ventricular morphometry in individuals with ASD, and ventricular changes were not examined through longitudinal follow-up. To better elucidate the relationship between ASD and brain ventricles, prospective longitudinal studies incorporating cerebrospinal fluid (CSF) analysis and more detailed ventricular assessments are needed.

### 4.3 Corpus callosum volumes in ASD

In our study, corpus callosum (CC) volumes were significantly higher in the ASD group compared to the TD group. A recent meta-analysis reported overlapping abnormalities in the corpus callosum tract during the transition from childhood to adulthood in individuals with ASD (25). In childhood ASD, reduced corpus callosum (CC) volumes have been reported in the context of abnormal white matter structure; however, age-related increases in CC volume have also been observed, which may be associated with difficulties in social communication and interaction (87). A systematic review reported that children under the age of six with ASD exhibited lower corpus callosum and cerebellar volumes compared to TDs, and that reduced CC volume was associated with hypoconnectivity between brain regions (88). A recent systematic review and meta-analysis reported reduced CC volume in children diagnosed with ASD, which has been attributed to atypical white matter structure and potentially linked to abnormal myelination (89). Another recent review reported reductions in corpus callosum white matter integrity in individuals with psychotic bipolar disorder compared to controls (90). A recent meta-analysis on ADHD reported volumetric reductions in the CC, which, similar to ASD, may be related to alterations in interhemispheric connectivity (91). In addition, it has been noted that changes in the CC are not limited to age-related variation but also exhibit sex-related differences, and that CC assessments should not rely solely on a single cross-sectional study but be supported by longitudinal follow-up studies (92). The corpus callosum alterations observed in our study may suggest that such morphometric changes can vary depending on age, sex, and the neuroimaging techniques used. To better clarify this finding, advanced longitudinal studies incorporating assessments of atypical white matter and myelination are needed.

The significant group differences observed in the morphometric parameters of the optic chiasm, along with the volumes of the choroid plexus, corpus callosum, and third ventricle, suggest that both commissural connectivity and specific neurodevelopmental deviations at the ventricular and optic pathway levels may co-occur in ASD. An integrated evaluation of these structures may enable the development of a more comprehensive, systems-level model to better explain the neurobiological basis of ASD.

### 4.4 Strengths and limitations

The strengths of our study include the inclusion of young children in both groups, the matching of groups for age and sex, and the consideration of confounding factors such as total brain volume, age, and sex. Morphometric analyses were conducted using a multivariate logistic regression model to predict autism diagnosis. Additionally, *post-hoc* power analyses were performed to assess the adequacy of the sample size for the morphometric analyses. To our knowledge, this is the first MRI-based study to investigate optic nerve and optic chiasm morphometry in children with ASD and to examine the impact of these morphometric differences on clinical severity in ASD.

However, our study has several limitations. ASD diagnoses were established based on clinical evaluation according to DSM-5 criteria, without the use of specialized assessment tools such as the Autism Diagnostic Observation Schedule (ADOS) or the Autism Diagnostic Interview (ADI). Neuroimaging data were obtained at a single time point, preventing analysis of the effects of comorbid psychiatric disorders, which may emerge during follow-up, on the brain regions studied. Changes in brain morphometric measurements over time related to sex and age were not examined, nor was the relationship between these changes and CSF symptom severity assessed through repeated measurements. Additionally, potential sex- and age-specific associations of regional measurements in ASD participants were not evaluated in our study. Specific IQ measurements and socioeconomic status, which could be potential confounding factors affecting the statistical results of the morphometric analyses, were not included as covariates in our study. Additionally, specific IQ scores for participants in both groups were not available. In our study, psychotropic medication use among participants with ASD could not be included as a covariate due to limitations in the data, and the impact of such medication use could not be examined in detail. This was a retrospective study utilizing previously acquired MRI data obtained for other purposes in the control group. As such, potential confounding variables and the lack of control over imaging parameters in typically developing participants could not be fully assessed in terms of their possible influence on the results. Additionally, future emergence of psychiatric conditions in control participants could not be evaluated due to the absence of longitudinal follow-up. Our study examined only macroscopic anatomical differences within a certain age range in both groups; measurements targeting retinal and choroidal changes, such as those obtained via optical coherence tomography (OCT), were not performed. Furthermore, advanced neuroimaging techniques such as functional MRI (fMRI) and cerebral blood flow assessments, which could evaluate functional connectivity and brain functionality, were not utilized in our morphometric analyses. This study utilized only structural MRI. Incorporating optical coherence tomography (OCT) and functional MRI (fMRI) in morphometric studies of ASD could provide better insight into how these structural differences affect brain function. Advanced studies including OCT, OCT-angiography, and fMRI are needed to more comprehensively assess structural brain function in individuals with ASD. While our study focused on macroscopic changes in the optic chiasm and optic nerve, actual visual function was not assessed. Had accurate and comprehensive visual testing been conducted, the functional capacity of optic nerve anatomy and retinal structures could have been directly measured, allowing correlations between anatomical measurements and functional outcomes. This limitation restricts our ability to clearly elucidate the impact of anatomical changes on visual function. Future research should address this limitation by investigating potential correlations between morphological parameters such as the optic nerve and optic chiasm and visual test outcomes. In our study, symptoms related to sensory sensitivities were assessed using the AuBC; however, other advanced tools for evaluating sensory sensitivities (such as the Sensory Profile 2) were not included. Another limitation of our study is that the instrument used was not specifically designed to assess sensory characteristics. In our study, differences between the right and left CP volumes were not examined, and lateralization could not be assessed. Moreover, morphometric measurements of the visual cortex could not be included in the current analysis. In terms of neuroimaging, our study evaluated only the morphometric levels of the optic nerve, optic chiasm, corpus callosum, choroid plexus, and brain ventricles. Additionally, our study focused exclusively on children and did not include adult participants. Neuroinflammation was not assessed, CSF analysis was not performed, and potential inflammatory variables could not be correlated with morphometric measurements in the context of ASD.

## 5 Conclusion

In summary, we found that children with ASD aged 5 to 13 years exhibited increased OC height levels, as well as larger CP and CC volumes, and decreased third ventricular volumes compared to TD children. These findings suggest that OC height, CC, CP, and third ventricular volumes may play a role in the etiopathogenesis of brain development in children with ASD. Additionally, multivariate logistic regression indicated that left CP volume was more strongly associated with ASD diagnosis than other morphometric differences. Significant structural variations in optic chiasm morphometry, along with the volumes of the choroid plexus, corpus callosum, and third ventricle, may reflect ASD-specific differences in neurodevelopmental trajectories. Integrating these parameters into combined biomarker profiles through multidisciplinary assessments could not only enhance early diagnostic approaches but also provide a structural reference framework for targeted interventions. Nevertheless, further research is needed to better understand the potential roles of these brain regions in ASD, to evaluate visual-sensory sensitivity through brain morphometric analyses, and to identify abnormal social-communicative behaviors using neuroimaging techniques.

## Data Availability

The original contributions presented in the study are included in the article/supplementary material. Further inquiries can be directed to the corresponding author.
